# Training of Classification Models via Federated Learning and Homomorphic Encryption

**DOI:** 10.3390/s23041966

**Published:** 2023-02-09

**Authors:** Eduardo Angulo, José Márquez, Ricardo Villanueva-Polanco

**Affiliations:** Department of Computer Science and Engineering, Universidad del Norte, Barranquilla 081007, Colombia

**Keywords:** training, federated learning, homomorphic encryption, classification models

## Abstract

With the rise of social networks and the introduction of data protection laws, companies are training machine learning models using data generated locally by their users or customers in various types of devices. The data may include sensitive information such as family information, medical records, personal habits, or financial records that, if leaked, can generate problems. For this reason, this paper aims to introduce a protocol for training Multi-Layer Perceptron (MLP) neural networks via combining federated learning and homomorphic encryption, where the data are distributed in multiple clients, and the data privacy is preserved. This proposal was validated by running several simulations using a dataset for a multi-class classification problem, different MLP neural network architectures, and different numbers of participating clients. The results are shown for several metrics in the local and federated settings, and a comparative analysis is carried out. Additionally, the privacy guarantees of the proposal are formally analyzed under a set of defined assumptions, and the added value of the proposed protocol is identified compared with previous works in the same area of knowledge.

## 1. Introduction

Machine learning techniques may solve data science problems via training models for prediction and classification. Thanks to the increase in the use of cloud resources, the training of these models is carried out on remote servers [1,2]. Consequently, data must be transferred remotely, which has increased concerns regarding its privacy since data may be leaked or misused [3]. Due to these concerns, multiple entities have introduced laws for data protection [4].

Advanced techniques (mainly cryptographic ones) are required to mitigate the risks related to data privacy; therefore, to securely perform the training of machine learning models, e.g., by making each participant only have access to a portion of the data or making the model not save sensible data. This set of techniques is enclosed within a new field known as private machine learning [5]. In practice, these techniques are combined since each has pros and cons, and a set of techniques can be adequate for a particular setting but not for any other.

Two widely known privacy-enhancing technologies are federated learning [6], which is a distributed machine learning approach that uses decentralized data and seeks to generate a model from data stored on multiple remote devices or clients, and homomorphic encryption [7,8] that is a form of cryptography in which mathematical or logical operations can be performed on encrypted data. In this work, both technologies are used to introduce a protocol to train multilayer perceptrons for classification while preserving data privacy during training.

This paper is organized as follows. Section 2 presents a literature review of the recent advances in private machine learning, concretely federated learning, and homomorphic encryption. Section 3 describes the approach and explains the problem to be solved by introducing the proposal. In addition, it defines the assumptions made, the hyperparameters, and the inner workings. Section 4 describes the methodology of the simulations and scenarios to validate the proposal. Section 5 analyzes the results of the experiments and provides an analysis of the privacy guarantees of the proposal. Finally, Section 6 provides conclusions and describes future research lines for continuing this work.

## 2. Previous Work

This section describes the recent works and advances made in the areas of federated learning and private machine learning.

Aono Y. et al. [9] present a method to perform the training of logistic regression on a server using data encrypted with a homomorphic cryptosystem with additive properties, under the assumption that such a server is honest but curious, with the aim of protecting the client’s training and test data.

To achieve their objective, the authors transform the logistic regression into an equivalent approach in which the previously mentioned homomorphic cryptosystem can be used. For training, the server receives the data encrypted with the client’s public key and performs the calculations to obtain the optimal values (minimizer) of the logistic regression. Finally, the server sends the minimizer to the client, who decrypts it using its private key.

The authors perform their solution experiments using two datasets from the UCI repository [10] and compare their performance against a logistic regression trained on unencrypted data based on accuracy, AUC, and F1 score metrics.

The first dataset used is Pima diabetes, where the approximation model trained with encrypted data presents the best performance in the three metrics. The second and last dataset is SPECTF heart disease, where the conventional model trained with unencrypted data performs better in the three metrics. It is followed by the approximation model trained with encrypted data. These results demonstrate that it is possible to train logistic regression models with encrypted data that present better or similar results than those trained while maintaining the security and privacy of the data used.

Hardy S. et al. [11] propose a solution to train a logistic regression model in a federated learning setup between two data providers and a coordinator, where data privacy is maintained. The data used are vertically partitioned, i.e., by features, and distributed between the two data providers.

To develop their solution, the authors assume a security scheme where the participants are honest but curious. Additionally, they use a homomorphic encryption technique with additive properties, specifically, the Paillier cryptosystem [12], to maintain the data privacy.

Due to the above, the authors need to adapt the training and the logistic regression model to work with the previously mentioned techniques. For this purpose, they approximate the logistic loss using the Taylor series (Taylor loss).

Several datasets from the UCI repository [10] are used during the experiments, converting them into binary classification problems if necessary. It is shown that the models trained with Taylor loss present, in most cases, equal or superior performance than those trained using logistic loss based on the accuracy and area under the curve (AUC) metrics.

Finally, the authors experiment with a quasi-real scenario using Kaggle’s “Give me some credit” dataset [13]. This proves that their solution can be scaled. In the same way, they demonstrate that the training performed using their solution is as good as or even better than conventional methods, with the disadvantage of requiring more resources (processing and time) during the training time.

Zheng H. et al. [14] perform the deployment of trained models using the federated learning (FL) technique to solve classification problems in mobile device scenarios by evaluating their performance, privacy, CPU consumption, and bandwidth consumption.

To achieve their goal, clients receive a set of training parameters, download the current model from the server, modify their local data to bring it into the correct form for their model input, and obtain the corresponding round update. Then, the server takes those updates and combines/averages them, thus obtaining the final updates; this process is repeated in a defined number of rounds.

The authors perform the experiments with three public datasets where they train a neural network with two hidden layers of 30 neurons, each with ReLU activation function and softmax output layer to perform classification. The datasets used are NYC Taxi [15], BR2000 [16] and Adult [10].

From the experiments, the authors conclude that the models trained using FL perform better with few clients, in addition to high CPU consumption on mobile devices where updates are calculated locally. However, they show low bandwidth consumption during communication with the server. Finally, concerning privacy, they conclude that the updates sent to the server are vulnerable to inferences, so they propose that this can be improved by applying secure aggregation methods.

Bonawitz K. et al. [17] propose a secure, low-cost-per-communication, robust-to-failure aggregation protocol in a mobile device configuration. This protocol uses various cryptographic systems for the necessary processes, including cryptographic key agreement, shared secret exchange, and authenticated encryption.

The authors present two variants: a more efficient one that is secure against attacks from an honest but curious adversary and a less efficient one that is secure against attacks from active adversaries. It is demonstrated by a security analysis that is performed on both variants.

From the analyses performed, the authors conclude that the proposed protocol is secure and maintains the privacy of the information since the server can only learn from the data after the aggregation process and not from the clients’ data.

## 3. Approach

This section describes the proposed approach and is organized as follows. Concretely, this section presents the description of the problem, the assumptions that guided the development of the approach, the hyperparameters used, and the methodology followed.

### 3.1. Problem Statement

Given a multi-class classification problem *C* and dataset *D* distributed among n_clients clients, the problem is to develop a protocol *P* by which the clients perform the joint training of MLP neural networks while preserving the data privacy. Preserving data privacy is meant that a participant in the protocol can only learn what it is intended to learn.

To verify the learning capability of an MLP neural network trained by the proposed protocol, a series of simulations training the model locally and distributively using the protocol *P* are performed. Then, a comparison between the resulting models is carried out, and an analysis of the privacy guarantees provided by the protocol is presented.

### 3.2. Assumptions

To develop the approach, the following assumptions are made:

- There is a main server *S* and clients $C_{1},\ldots ,C_{n\_clients}$, with n_clients≥1. The server will serve as an aggregator and distributor of some data. In contrast, the clients will serve as computational endpoints, i.e., each client will train models over local data combined with some extra data received from other participants.
- All participants have the computational capacity to perform all the necessary operations.
- The main server can establish a communication channel with any other client $C_{i}$, 1≤i≤n_clients (via TCP, for example). Similarly, any client $C_{i}$ can establish a communication channel with any other client or the server *S* (via TCP, for example).
- The participants are assumed to form a ring topology, i.e., the client $C_{1}$ connects with $C_{2}$, which connects with $C_{3}$, and so on to the last client $C_{n\_clients}$. This last client connects with the main server *S*, which may connect with any other client.

Additionally, the assumed threat model is the honest but curious model, a well-known and accepted model in the multiparty computation (MPC) literature [18]. Concretely, the following is assumed:

- The participants (server and the clients) are assumed to be honest but curious.
- Each client has access to a portion of a specified dataset and uses it on the same number of features. Note that, for our experiments, each client uses a portion of a specified dataset divided equally by rows on the same number of features.
- After establishing a communication channel via TCP, for example, any two participants build a secure channel via an appropriate cryptographic protocol (such as TLS) over which they further exchange messages.

### 3.3. Hyperparameters

Before training a neural network, a set of hyperparameters is defined. These hyperparameters will be used by both the clients and the server:

- Number of input features (n_features).
- Number of output classes (n_classes).
- Number of neurons per hidden layer (hidden_layers_size).
- Activation Functions per hidden layer (activations).
- Initialization method for weights and biases (initialization).
- Learning rate γ (learning_rate).
- Number of epochs (epochs).
- Method for preprocessing input data (standardization / normalization)
- Codification of the output (encoding).
- An additively homomorphic encryption scheme E=(G,E,D) that consists of a triple of efficient algorithms: a key generation algorithm *G*, an encryption algorithm *E*, a decryption algorithm *D* [7,19].
  – *G* is a probabilistic algorithm that is invoked as $\left(pk,sk\right)\overset{R}{\leftarrow }G\left(\lambda \right)$, where λ is a security parameter, pk is called a public key and sk is called a secret key.
  – *E* is a probabilistic algorithm that is invoked as $c\overset{R}{\leftarrow }E\left(pk,m\right)$, where pk is a public key (as output by *G*), *m* is a message, and *c* is a ciphertext.
  – *D* is a deterministic algorithm that is invoked as m←D(sk,c), where sk is a secret key (as output by *G*), *c* is a ciphertext, and *m* is either a message or a special reject value (distinct from all messages).
  – It is required that decryption undoes encryption, specifically for all possible outputs (pk,sk) of *G*, and all messages *m*,
    Pr[D(sk,E(pk,m))=m]=1.
    holds.
  – Messages are assumed to lie in some finite message space M, and ciphertexts in some finite ciphertext space C. So E=(G,E,D) is defined over (M,C).
  – Homomorphic Property: there is a binary operator ⋄ such that for ciphertexts $c_{0}\overset{R}{\leftarrow }E\left(pk,m_{0}\right)$ and $c_{1}\overset{R}{\leftarrow }E\left(pk,m_{1}\right)$, $c\leftarrow c_{0}⋄c_{1}$ is an encryption of $m_{0}+m_{1}$ ( the sum of the underlying plain texts).

The implementation of the proposed protocol uses the Paillier encryption scheme:

- List of all domain names or IP addresses of the clients and the server.
- The security parameter λ is configured only on the server to let it generate the key pair (pk,sk) for the homomorphic encryption scheme; pk is published so each client has access to it, while sk is securely stored in the main server.

### 3.4. Inner-Working of the Approach

This section describes the inner workings of the proposal.

**Figure 1:**
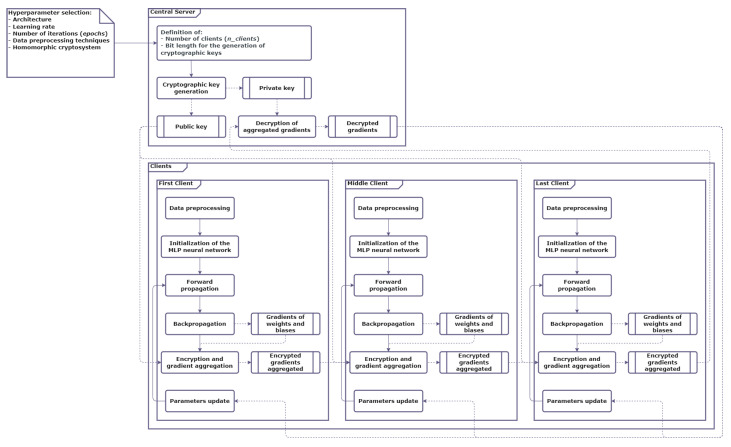
Inner-working of the proposed protocol.

1. Each client performs the data preprocessing locally and instantiates its neural network based on the configured hyperparameters, resulting in each client having the same neural network architecture. Each client then initializes the neural network’s weights and biases according to the configured method.
2. Simultaneously, the main server uses the security parameter to generate a key pair, i.e., the public key and the corresponding private key, for the configured homomorphic encryption scheme. The public key is published so that each client has access to it.
3. The client $C_{1}$ proceeds with training its neural network over its local data as per the configured number of epochs. This process consists of performing the forward and backward propagation and obtaining the gradients for the weights and biases. These gradients are encrypted with the server’s public key and then are sent to the next client $C_{2}$. Consequently, $C_{2}$ proceeds with training its neural network over its local data as per the configured number of epochs. Once this process ends, $C_{2}$ will obtain the gradients for the weights and biases, which are encrypted with the server’s public key and aggregated (homomorphic summed) to the encrypted gradients received previously. This aggregate is then sent to the next client, $C_{3}$. The remaining clients follow the same rules, except the last one, $C_{n\_clients}$, which sends its encrypted aggregate (containing the sum of all gradients) to the main server, *S*.
4. The main server decrypts the received aggregate with its private key, then calculates the average for each gradient and sends all of them to the clients. Each client then updates its corresponding neural network’s trainable parameters with the aggregate sent by the server, i.e., $X_{n+1}=X_{n}-\gamma \nabla F\left(X_{n}\right),\;n\geq 0$, for each trainable parameter X.
5. The steps 3 and 4 are repeated as per the number of epochs. This process is depicted in Figure 1.

## 4. Experiments

### 4.1. Simulation

The validation of the protocol is achieved by performing a series of simulations. As a remark, the number of samples per client (m_client) is given by Equation (1).

(1) $$m\_client=\left\lfloor \frac{(1-test\_size)\times m}{n\_clients}\right\rfloor$$

where m=|D| is the number of samples of the dataset used, and test_size is the portion of the data taken to validate the MLP network.

#### 4.1.1. Dataset Selection and Analysis

**Figure 2:**
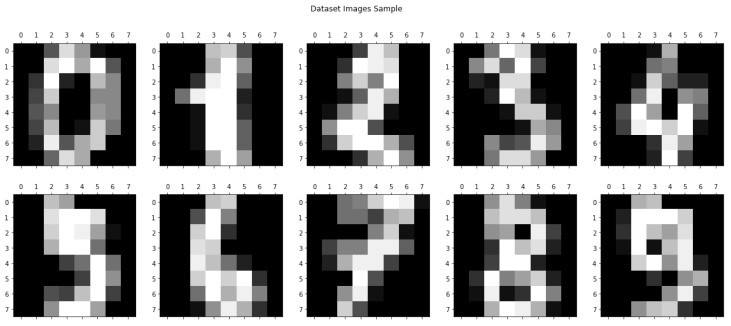
Dataset images sample.

Based on the problem of this research, the dataset used was the “Optical Recognition of Handwritten Digits Dataset” sample from UCI [10] since data represent a multiclass classification problem that can be solved by using MLP networks and on which the proposed protocol can be applied. These data were provided by the scikit-learn library [20] using the load_digits function that gives us the data as Numpy arrays. This dataset has a total of *m* = 1797 samples, where each sample has 64 input features, and the values they take are integers in the range [0, 16]. Each of these values represents the color of a pixel of an 8 × 8 grayscale image, with zero (0) being the color black and sixteen (16) being the color white. Figure 2 shows a sample of the images representing the data.

**Figure 3:**
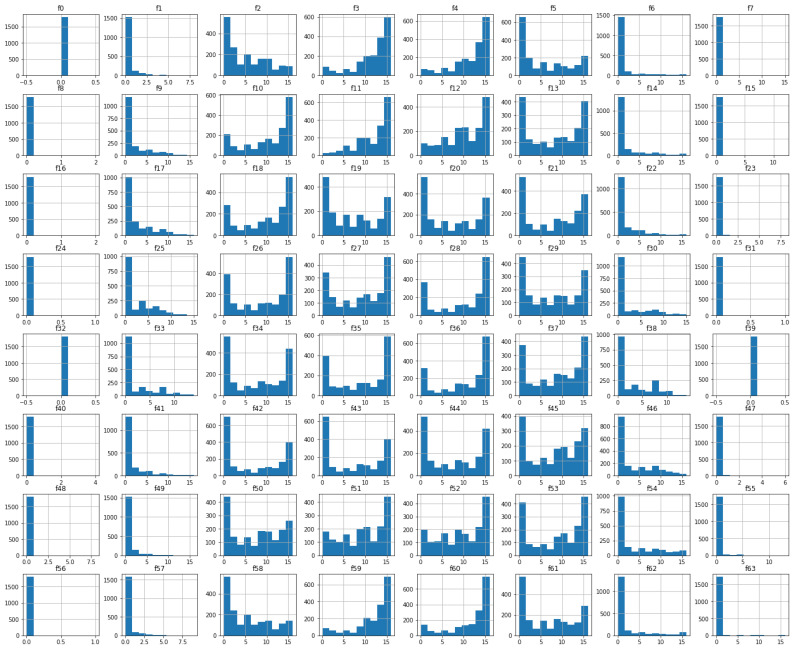
Feature distribution of the original dataset.

No NaN values or outliers were found in the data obtained since they were all within the range mentioned above. Their distribution can be seen in Figure 3.

The target variable defined for this dataset corresponds to the digits (from 0 to 9) of the input data/images, indicating ten output classes. The distribution of these classes can be seen in Table 1.

#### 4.1.2. Data Preproccessing

Since the dataset has no NaN values or outliers, it is not necessary to remove any of the samples from it. Therefore, the subsequent processes will be carried out with all the previously mentioned samples, i.e., 1797.

The model to be developed is an MLP neural network for multiclass classification. Therefore, the target variable is encoded by using the One Hot technique to take the values to several columns equal to the number of classes, where the value in a column is one (1) if it represents the corresponding class or zero (0) in any other case. This method was selected because the output layer has a softmax function and requires the data in this form for its correct operation. The method to perform this encoding was provided by the scikit-learn library [20].

Subsequently, preliminary tests were performed to verify the correct operation of the MLP networks. Then, it was necessary to scale the data because, in some tests, the model diverged. This work was carried out by applying the Standard scaling method, which uses the features’ mean and standard deviation to perform the features’ scaling. The method to perform this scaling was provided by the scikit-learn library [20].

Applying the scaling and performing the same preliminary tests resulted in the previous models that diverged coming to converge. Those that converged showed better results because the range of the characteristics was the smallest. The expected behavior is evident after applying the standard scaling method since the data distribution remains the same within the corresponding data ranges.

Finally, and as shown by Table 1, the classes of the target variable are sufficiently balanced, where the class with the highest number of samples has 183 and the one with the lowest number of samples has 174, showing a difference of nine samples between them. Therefore, applying any method for balancing the classes is optional.

#### 4.1.3. Hyperparameters Selection and Design of Scenarios

In addition to the preliminary tests for selecting data preprocessing methods, tests were performed with different numbers of clients (n_clients), training only with data locally, to determine the most appropriate set of hyperparameters to use in the simulations.

The following constant values are set to non-varying hyperparameters per test:

- The size of the test set, test_size, was set to 0.1, meaning that 10% of the dataset samples are taken to perform the model validations.
- The homomorphic cryptosystem is set to the Paillier cryptosystem. Its implementation is provided by the python-paillier library [21].
- The length in bits to generate the cryptographic keys for the Paillier cryptosystem key_length was set to 1024 bits.
- The learning rate learning_rate was set to 0.01 since preliminary tests confirmed that it is a good value for training purposes.
- The number of iterations epochs was set to 120, which was confirmed similarly as learning rate was.

Some tests are performed to adjust the number of hidden layers and the number of neurons in them (hidden_layers_size), the activation functions of the hidden layers (activations), and the initialization method of the trainable parameters (weights and biases). Note that the number of trainable parameters for the defined networks is given by Equation (2).

(2) $$n_{params}=\sum_{i=1}^{n_{layers}-1}n^{\left[i\right]}\times \left(n^{\left[i-1\right]}+1\right)$$

where $n_{layers}$ is the total number of layers of the neural network, $n^{\left[i\right]}$ is the number of neurons in layer *i*, and $n^{\left[0\right]}$ is the number of features in the dataset.

As a result of running these tests, three scenarios, described below, were set up to measure the impact of the number of clients and the number of tractable parameters on the training and execution times of the proposed protocol, as well as the efficiency of the protocol under the various configurations to verify that the protocol works in a generalized way for MLP networks.

**Scenario 1**

**Figure 4:**
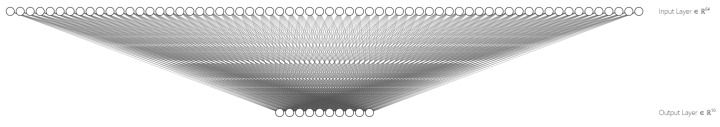
Graphical representation of the MLP neural network of scenario 1.

The network has no hidden layers for this scenario, so the only varying hyperparameter is the initialization method. At first, some tests were performed with five clients to infer that the best method for initializing the trainable parameters is zero initialization. The results of the tests using zero initialization outperformed the results of the tests using the random and initialization methods according to the metrics obtained from the test data. The resulting MLP neural network can be seen in Figure 4.

**Scenario 2**

**Figure 5:**
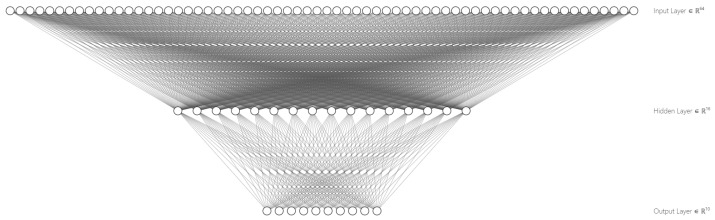
Graphical representation of the MLP neural network of scenario 2.

This scenario consists of a neural network having a single hidden layer. For this scenario, the varying hyperparameters are the initialization method, the number of neurons, and the activation function of the hidden layer. By doing a hyper-parameter exploration with four clients for initialization∈{random,zero,he}, hidden_layers_size∈{2,4,6,16,32} and activation∈{tanh,sigmoid,relu}, the combination consisting of initialization=he,hidden_layers_size=16 and activation=tanh outperformed the others. The resulting MLP neural network can be seen in Figure 5.

**Scenario 3**

**Figure 6:**
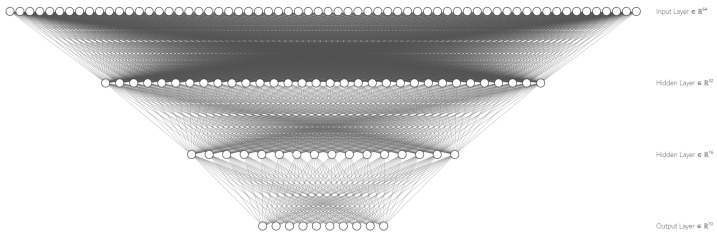
Graphical representation of the MLP neural network of scenario 3.

For this scenario, the neural network has two hidden layers. The varying hyperparameters are the initialization method, the number of neurons, and the activation function of the hidden layers. By performing a hyper-parameter exploration with with three clients for initialization∈{random,zero,he}, hidden_layers_size∈{2,4,6,16,32} and activation∈{tanh,sigmoid,relu}, the combinations outperforming the others resulted to be those with initialization=he, hidden_layers_size={16,32} and activation=tanh. The resulting MLP neural network can be seen in Figure 6.

**Scenarios Summary**

Table 2 shows a summary of the relevant information and hyperparameters of the three scenarios defined.

## 5. Results Analysis

This section will describe the environment in which the simulations were run for the scenarios defined in the previous section, show the results of these runs using the metrics of accuracy, loss (given by cross-entropy), sensitivity, precision, and ROC-AUC and analyze them from the federated learning point of view.

### 5.1. Scenarios Evaluation

All simulations for the protocol verification were executed on a single computer whose specifications are mentioned in Section 5.1.1. The computer simulates a computer network where data is passed directly between the simulated entities (clients and central server) without using sockets. The above means that the communication overhead is considered to be negligible.

For each proposed scenario, two experiments are performed. The first experiment refers to training the neural networks of the clients using only their local data (a portion of the dataset) without sharing any information (data, parameters, gradients of the parameters) with the other entities. In contrast, the second experiment uses the proposed protocol to train the neural networks in each client, after which the encrypted gradients are transmitted to the other clients in series, carrying out their aggregation until they reach the server. Finally, the server can decrypt it and calculate the updates of the parameters of the corresponding epoch.

#### 5.1.1. Execution Environment

The simulations of the previously defined scenarios were run on a Dell computer with Ubuntu 18.04.5 LTS operating system, 8 GB of RAM, a 1.80 GHz Intel Core i7-10510 processor with eight cores, and 512 GB of solid state hard disk (SSD).

The simulations were performed using the Python programming language in version 3.8.13, where a jupyter notebook was used to record the results. Additionally, *numpy*, *scikit-learn*, *python-paillier*, *matplotlib*, and *seaborn* libraries were used during the development of the simulations.

#### 5.1.2. Scenario 1

**Figure 7:**
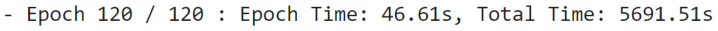
Protocol execution times in scenario 1.

The scenario was run using the specifications mentioned in the previous section. The execution times per epoch and total protocol are evidenced in Figure 7.

Table 3 shows the metrics (accuracy, loss, precision, recall, and ROC-AUC) for the five clients in this scenario when training using the local data and applying the proposed federated learning protocol, as well as their average across both training sessions.

As can be seen in the results presented in Table 3, all the metrics improve (increase in the case of accuracy, precision, sensitivity, and ROC-AUC or decrease in the case of loss) or remain the same when training using the proposed protocol, except precision for client 1.

These results for the metrics obtained are within the expected since the training, through the protocol, is performed using information from all clients, thus increasing the number of samples used in training.

The accuracy for the predictions is very high for all the classes belonging to the dataset, except for class 9, where the highest number of failures is found for all the clients.

#### 5.1.3. Scenario 2

**Figure 8:**
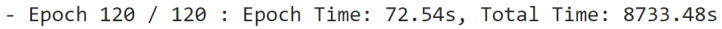
Protocol execution times in scenario 2.

The scenario was run using the specifications mentioned in the previous section. The execution times per epoch and total protocol are evidenced in Figure 8.

Table 4 presents the metrics (accuracy, loss, precision, recall, and ROC-AUC) for the four clients in this scenario when training using the local data and applying the proposed federated learning protocol, as well as their average for both training sessions.

As can be seen in the results presented in Table 4, all the metrics obtained by running the proposed protocol for clients 1 and 4 show an improvement for training with local data, while for client 2, all the metrics improve except the ROC-AUC. However, for client 3, all metrics worsen when running the proposed protocol, although the average of each of the metrics for the proposed protocol is better than the corresponding average for the training where only local data was used.

The results obtained for client 3 are because this client outperforms the other clients (given by the metrics) when only local data is used during training. When the protocol is run, the noise generated by the other clients is introduced, thus resulting in the training and metrics for this client getting worse.

It is worth noting that the metrics are worse than those presented in the first scenario. The above suggests that the architecture used in this scenario could be better for this dataset. However, results are obtained about the calculated metrics within the expected range due to the use of information from all clients, i.e., increasing the number of samples used in training through the protocol.

The degree of accuracy of predictions is high for most of the classes belonging to the dataset. However, some classes present high levels of prediction failure, and these differ from client to client; for client 1, they are classes 0 and 8; for client 2, they are classes 4, 8, and 9; for client 3, they are classes 1 and 8 and; for client 4, they are classes 1, 4 and 9, where they tend to improve on training with the protocol.

#### 5.1.4. Scenario 3

**Figure 9:**
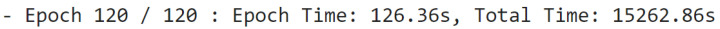
Protocol execution times in scenario 3.

The scenario was run using the specifications mentioned in the previous section. The execution times per epoch and total protocol are evidenced in Figure 9.

Table 5 presents the metrics (accuracy, loss, precision, recall, and ROC-AUC) for the three clients in this scenario when training using the local data and applying the proposed federated learning protocol, as well as their average for both training sessions.

As shown by the results presented in Table 5, all the metrics obtained by running the proposed protocol for clients 1 and 3 show an improvement in training with local data. However, for client 2, all metrics worsen when running the proposed protocol, although the average of each of the metrics for the proposed protocol is better than the corresponding average for the training where only local data was used.

The results obtained for client 2 are because this client outperforms the other clients (given by the metrics) when only local data is used during training. When the protocol is executed, the noise generated by the other clients is introduced, thus resulting in the training and metrics for this client getting worse.

The metrics obtained in this scenario are worse than those obtained in the second scenario and, therefore, worse than those obtained in the first scenario. With these results and those of the previous scenario, it can be concluded that architectures with higher hidden layers obtain worse metrics for this dataset. However, the metrics’ results are obtained within the expected range thanks to the training performed with the protocol, which uses a more significant number of samples corresponding to all the clients’ information.

The accuracy for predictions is high for some classes belonging to the dataset, these being the worst performers throughout all scenarios. However, several classes show very high levels of failure in the prediction, in some cases completely missing a class, where these differ from client to client; for client 1, they are classes 0, 3, 5, and 8; for client 2, they are classes 1, 3, and 9 and; for client 3, they are classes 1, 2, 3, 5, and 8, where they tend to improve in the training with the protocol.

### 5.2. Discussion

The contribution of this paper is a protocol to perform MLP neural network training for classification using a federated learning scheme. In the protocol, the privacy of the client data is preserved, and better results are presented on average concerning performing the training using only the local data held by each client.

#### 5.2.1. Learning Capability

Compared to proposals studied during the literature review, the protocol proposed in this research generalizes the training of MLP neural networks using the federated learning scheme compared to the logistic regression models proposed by other authors. Additionally, the proposed protocol allows training with multiple clients without exposing their data to the other participants of the protocol as opposed to other works, where only two clients are trained and transmit their encrypted data to a third party. Finally, the proposed protocol uses a secure aggregation method based on homomorphic encryption, while other authors propose methods for the same purpose without using this type of encryption.

**Figure 10:**
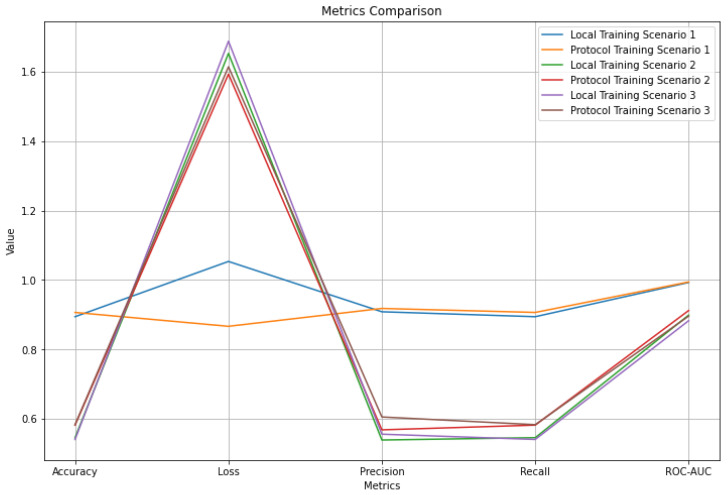
Comparison of average metrics for all scenarios.

Regarding the training, using the proposed protocol, on average, the evaluation metrics improve because, ultimately, the training is performed using data from all clients. The above can be observed in Figure 10 for each pair of metric sets of the scenarios. However, it is essential to mention that there are cases where the metrics worsen for a specific client whose local model significantly outperforms others. The latter is because the model trained using the protocol includes the noise generated by other clients, making said model worse than the one trained locally for this client.

Additionally, it is noted that the metrics presented for scenario 1 are much better than those presented for scenarios 2 and 3. In contrast, scenarios 2 and 3 do not differ much in their metrics, some being higher in scenario 2 and others in scenario 3.

**Figure 11:**
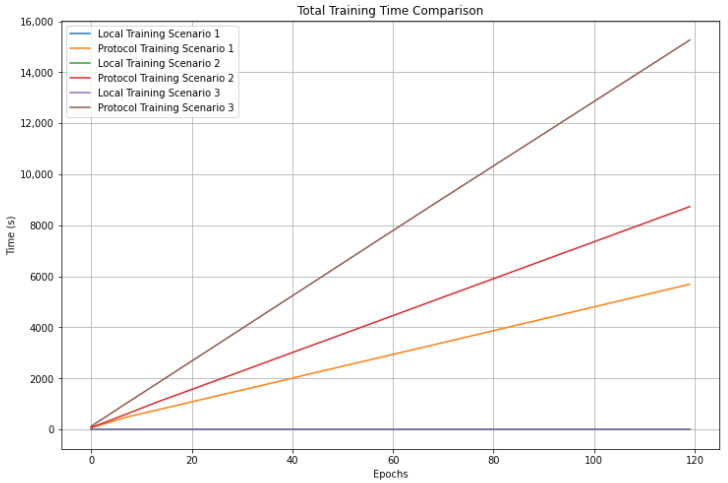
Comparison of training times for all scenarios.

Another aspect to mention is that the execution times of the proposed protocol are directly influenced by the number of clients participating in the training and by the number of parameters to be deduced. The times are considerably higher than those of training with local data because the processes of encryption, decryption, and homomorphic operations have a high computational cost. It can be seen in Figure 11, which shows the comparison of local and protocol training times for all three scenarios.

**Figure 12:**
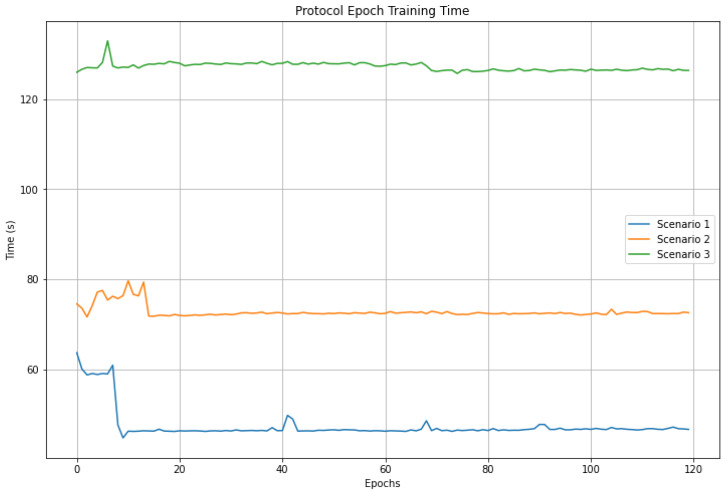
Comparison of training times by epoch using the training protocol.

**Figure 13:**
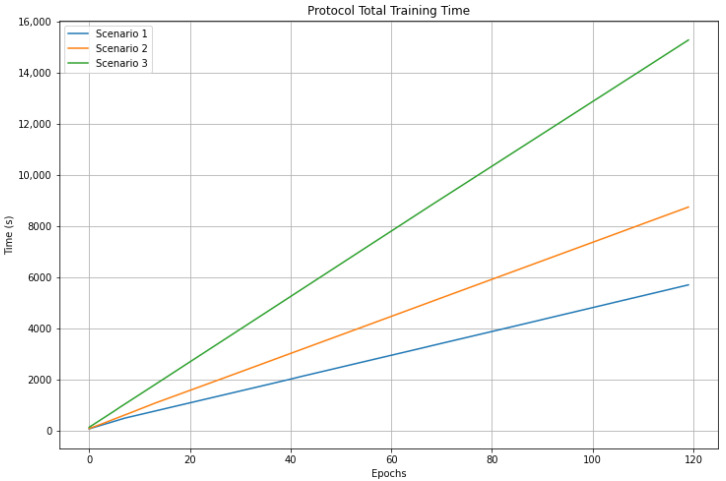
Comparison of the total training times using the protocol.

Likewise, Figure 12 and Figure 13 show the times per epoch and total training times using the protocol for the three scenarios, where it can be seen that the times presented in scenario 1 are lower than in scenario 2. In turn, these are lower than in scenario 3. These results show the impact of the number of trainable parameters and clients on the training times due to the mentioned processes.

Since the amount of data used in the simulations is small, it does not significantly impact the processes that depend on it (forward propagation and backpropagation), so its impact on the training times is considered negligible. Regarding the number of clients, this linearly affects the training times of the protocol because each client must perform the encryption and gradient aggregation operations (except for the first one that only performs gradient encryption), and all clients were simulated in the same computer. Similarly, the number of parameters is the variable that affects the training times. Because of this number, the gradient encryption and aggregation operations must be performed, taking into account the time consumed by the selected homomorphic cryptosystem, i.e., Paillier.

The Equation (3) shows the training time given the number of clients $n_{clients}$ and the number of parameters $n_{params}$, where $avg_{T_{enc}}$ is the average encryption time and $avg_{T_{agg}}$ is the average aggregation time.

(3) $$T\left(n_{clients},n_{params}\right)=n_{clients}*n_{params}*avg_{T_{enc}}+\left(n_{clients}-1\right)*n_{params}*avg_{T_{agg}}$$

#### 5.2.2. Privacy Guarantees

Regarding data privacy, the passive attacks described by Boenisch F. et al. [22] are considered. In this regard, the proposal preserves data privacy in the honest-but-curious model:

1. A client never exposes its data to any other participant during the execution of the protocol since the client’s end device carries out all computations locally.
2. Note that a client receives the encrypted gradients from the previous client. Since the client does have access to the server’s private key, it cannot decrypt the encrypted gradients.
3. The server receives the encrypted aggregated gradients from all the clients, and even though it can decrypt them, it can not infer any data or features of any client’s dataset from them. Note that the server can not distinguish what portion of the aggregated gradients corresponds to which client. It is worth mentioning that data privacy might be compromised only if:
  - For a particular trainable parameter X, n_clients−1 out of n_clients gradients are computed as zero. In such a case, the server might use the non-zero gradient to infer data or reconstruct some feature from the corresponding client’s dataset, assuming the server can detect when the case occurs.
    Let us assume that $p_{X}^{i}$ is the probability that the gradient for the trainable parameter X computed by the client *i* is zero during an iteration of the protocol. Let $p=max\{p_{X}^{i},1\leq i\leq n\_clients\}$. Given that each client computes its training independently, the experiment of having *a* out of n_clients gradients for the trainable parameter X computed as zero follows a binomial distribution with probability *p*. Therefore, the probability of having n_clients−1 out of n_clients gradients for the trainable parameter X computed as zero is given by Pn_clients−1=n_clientsn_clients−1pn_clients−1(1−p)=(n_clients)pn_clients−1(1−p). Therefore, $P_{n\_clients-1}$ tends to zero as the number of clients increases for any 0≤p≤1. Moreover, note that if *p* is negligible, then $P_{n\_clients-1}$ is negligible too.
    Note that the server might analyze the function $np^{n-1}\left(1-p\right)$ as *n* increases and use a proper estimation for *p*. However, an accurate estimation for *p* is tough since *p* relies on each client’s dataset and the initial/updated values for the trainable parameter X. Therefore, this situation is hardly exploitable. Note that the server might always try to obtain some information from the aggregated gradient for a trainable parameter X and might succeed if the event occurred (which is negligible).
  - There exists a single client running the protocol. In such a case, the server might exploit this to obtain relevant information from the gradients sent by the client. However, this scenario is unrealistic since if there is a single client, such a client would prefer local training on its data without relying on the main server. Hence, and without loss of generality, the number of clients is assumed to be greater than one.

## 6. Conclusions and Future Work

This work proposed a cryptographic protocol to train MLP neural networks by combining federated learning and homomorphic encryption.

The efficiency and effectiveness of the proposed protocol are measured by running simulations and using the dataset “Optical Recognition of Handwritten Digits Dataset” provided by the scikit-learn library. Moreover, the privacy guarantees of the proposal were analyzed by looking at various attacks targeting the federated learning scheme under a set of defined assumptions.

Regarding the effectiveness of the protocol, by executing the proposed protocol, better values are obtained on average for most metrics in several clients. For those clients whose local training exceeded their metrics to a large extent those of the others, their metrics through the protocol tended to worsen.

Concerning the privacy guarantees, the analysis shows that the proposed protocol strives to preserve data privacy through the secure aggregation method based on homomorphic encryption since it prevents information leakage from client gradients.

The added value of this work lies in presenting a generalized protocol for training any MLP neural network with any number of clients under a federated learning scheme, as opposed to the training of logistic regression models with the number of clients limited to two, as recently presented works.

For future work, three research lines are worth exploring. The first would be adjusting our protocol to use a fully homomorphic cryptosystem and include regularization and optimization methods when training MLP neural networks. The second would be eliminating the central server, which is itself a point of failure. That would ideally mean the decryption and distribution of aggregated weights would be computed by all clients together. Finally, it would be interesting to implement the protocol in a private computer network with a less controlled environment, where the amount of data held by each participating client is different, and the processing capabilities are also potentially varying. 

## Figures and Tables

**Table 1:** Output classes distribution.

Class	Occurrences in the Dataset	Percentage of Total
0	178	9.905%
1	182	10.128%
2	177	9.850%
3	183	10.184%
4	181	10.072%
5	182	10.128%
6	181	10.072%
7	179	9.961%
8	174	9.683%
9	180	10.017%

**Table 2:** Summary of scenarios parameters.

Scenario	n_features	n_classes	hidden_layers_size
1	64	10	()
2	64	10	(16)
3	64	10	(32, 16)
activations	initialization	output_activation	trainable_parameters
()	zeros	softmax	650
(tanh)	he	softmax	1210
(tanh, tanh)	he	softmax	2778
cryptosystem	key_length	n_clients	training_size
Paillier	1024	5	90%
Paillier	1024	4	90%
Paillier	1024	3	90%
samples_per_client	learning_rate	epochs	test_size
323	0.01	120	10%
404	0.01	120	10%
539	0.01	120	10%

**Table 3:** Model training results for scenario 1.

Client	Train	Accuracy	Loss	Precision	Recall	ROC-AUC
1	Local	0.9167	1.0591	0.9265	0.9167	0.9935
	Federated	0.9167	0.8699	0.9244	0.9167	0.9949
2	Local	0.8722	1.0349	0.8873	0.8722	0.9929
	Federated	0.8778	0.8539	0.8914	0.8778	0.9944
3	Local	0.8833	1.0723	0.9140	0.8833	0.9928
	Federated	0.9111	0.8831	0.9322	0.9111	0.9938
4	Local	0.9000	1.0552	0.9047	0.9000	0.9929
	Federated	0.9222	0.8655	0.9285	0.9222	0.9949
5	Local	0.9000	1.0463	0.9104	0.9000	0.9932
	Federated	0.9056	0.8612	0.9143	0.9056	0.9946
Average	Local	0.8944	1.0536	0.9086	0.8944	0.9931
	Federated	0.9067	0.8667	0.9182	0.9067	0.9945

**Table 4:** Model training results for scenario 2.

Client	Train	Accuracy	Loss	Precision	Recall	ROC-AUC
1	Local	0.5167	1.7511	0.5032	0.5167	0.8612
	Federated	0.6167	1.6268	0.6047	0.6167	0.8963
2	Local	0.4944	1.7007	0.4857	0.4944	0.8852
	Federated	0.5056	1.6987	0.4992	0.5056	0.8846
3	Local	0.6556	1.5534	0.6747	0.6556	0.9356
	Federated	0.6167	1.5548	0.6333	0.6167	0.9328
4	Local	0.5167	1.6077	0.4927	0.5167	0.9145
	Federated	0.5889	1.4931	0.5366	0.5889	0.9354
Average	Local	0.5458	1.6532	0.5391	0.5458	0.8991
	Federated	0.5820	1.5934	0.5684	0.5820	0.9123

**Table 5:** Model training results for scenario 3.

Client	Train	Accuracy	Loss	Precision	Recall	ROC-AUC
1	Local	0.4944	1.6577	0.4959	0.4944	0.8967
	Federated	0.5889	1.5359	0.6342	0.5889	0.9197
2	Local	0.5889	1.7016	0.6510	0.5889	0.8865
	Federated	0.5722	1.7278	0.6215	0.5722	0.8769
3	Local	0.5389	1.7054	0.5209	0.5389	0.8658
	Federated	0.5889	1.5798	0.5608	0.5889	0.8920
Average	Local	0.5407	1.6882	0.5559	0.5407	0.8830
	Federated	0.5833	1.6145	0.6055	0.5833	0.8962

## Data Availability

The dataset used was the “Optical Recognition of Handwritten Digits Dataset” sample from UCI [10].
